# Magnetic resonance imaging findings in 46 elbows with a radial head fracture

**DOI:** 10.3109/17453674.2010.483988

**Published:** 2010-05-21

**Authors:** Laurens Kaas, Jeroen L Turkenburg, Roger P van Riet, Jos P A M Vroemen, Denise Eygendaal

**Affiliations:** ^1^Department of Orthopaedic Surgery; ^2^Orthotrauma Research Center Amsterdam, Department of Orthopaedic Surgery, Academic Medical Center, Amsterdamthe Netherlands; ^3^Department of Radiology; ^4^Department of Orthopaedic Surgery, Monica Hospital, AntwerpBelgium; ^5^Department of General Surgery, Amphia Hospital, Breda

## Abstract

**Background and purpose:**

Radial head fractures are common, and may be associated with other injuries of clinical importance. We present the results of a standard additional MRI scan for patients with a radial head fracture.

**Patients and methods:**

44 patients (mean age 47 years) with 46 radial head fractures underwent MRI. 17 elbows had a Mason type-I fracture, 23 a Mason type-II fracture, and 6 elbows had a Mason type-III fracture.

**Results:**

Associated injuries were found in 35 elbows: 28 elbows had a lateral collateral ligament lesion, 18 had capitellar injury, 1 had a coronoid fracture, and 1 elbow had medial collateral ligament injury.

**Interpretation:**

The incidence of associated injuries with radial head fractures found with MRI was high. The clinical relevance should be investigated.

## Introduction

Fractures of the head of the radius are common and account for approximately one-third of all fractures of the elbow. The outcome of undisplaced or minimally displaced radial head fractures is good (van Riet et al. 2009). Radial head fractures are usually classified according to the Mason-Hotchkiss classification into types I to III. A type-I fracture is minimally displaced (< 2 mm), a fracture with > 2 mm dislocation is a type-II fracture, and type-III fractures are comminuted (Hotchkiss 1997).

Recent studies have revealed a high incidence of associated injuries of the ipsilateral upper extremity with radial head fractures (Itamura et al. 2005, van Riet et al. 2005, Kaas et al. 2008, Nalbantoglu et al. 2009). Ligamentous and chondral injuries especially may go undetected by conventional radiographs, but may be important for treatment (Davidson et al. 1993, Steinmann, 2008, Nalbantoglu et al. 2009, van Riet et al. 2009). To assess the incidence of these injuries, we included a MRI scan of the elbow in patients with a radial head fracture and now we present our findings for the first 46 elbows.

## Patients and methods

44 patients (mean age 45 (20–75) years, 25 females) with 46 radial head fractures who presented with a radial head fracture at our emergency department within 48 h of trauma, and who were available for follow-up at our hospital, underwent an MRI scan of the elbow. 2 patients had bilateral fractures and 26 fractures were on the dominant side. Anteroposterior and lateral conventional radiographs were evaluated for associated osseous injuries of the ipsilateral upper extremity. Additional images such as oblique views and shoulder or wrist images were obtained when indicated.

An MR scan of the injured elbow was done 7 (1–16) days on average after injury. 10 other patients with radial head fractures did not receive an MRI scan, as the timing of it would adversely delay the treatment of the injury. 5 patients had a Mason type-II fracture (including 1 patient with a Monteggia lesion) and 5 patients had a Mason type-III fracture (including 1 patient with an olecranon fracture and posterior dislocation, 1 patient with an olecranon fracture and a type-III coronoid fracture after a posterior dislocation, and 1 patient with a coronoid fracture and posterior dislocation).

MR imaging was performed with a 1.5 Tesla scanner with a dedicated small flex coil. Patients were imaged in the supine position with the arm overhead and with the forearm supinate. Imaging began about 10 cm above the elbow joint and extended to the bicipital tuberosity. Images were acquired in the axial, coronal, and sagittal planes. Imaging comprised axial and coronal T1-weighted spin-echo (TR range/TE, 400-480/14) coronal fat-suppressed proton density-weighted fast spin-echo (TR/TE, 3500/30), coronal T2*-weighted gradient echo (TR/TE, 540/10), and sagittal T2-weighted fast-recovery fast spin-echo (TR/TE, 6000/67) sequences. The T1-weighted sequences were obtained with the following parameters: 12–14-cm field of view, 256 × 192 image matrix, and 3.2-mm section thickness with a 0.3–0.5-mm intersection gap. The T2*-weighted sequence was obtained with the following parameters: 12-cm field of view, 256 × 192 image matrix, and 3.2-mm section thickness with a 0.3-mm intersection gap. The T2-weighted sequence was obtained with the following parameters: 12-cm field of view, 256 × 192 image matrix, and 3-mm section thickness with a 0.3-mm intersection gap. 2 signals were acquired for all sequences.

The quality of the MR images was good in all but 2 elbows, where evaluation was difficult because of movement artefacts. The MRI scans and radiographs were evaluated by 1 of 2 experienced radiologists using a standardized scoring list. Osseous, chondral, and ligamentous injury or dislocation of the ipsilateral upper extremity in combination with a radial head fracture was regarded as an associated injury. Specific attention was given to: loose bodies, bone bruising or fracture, osteochondral damage, injury to the lateral collateral ligament (LCL) complex, common extensor tendon, medial collateral ligament (MCL) complex, common flexor tendon, and injury of the biceps and triceps tendon. Ligamentous injuries were divided into 4 subtypes: distortion (edema of the ligament, but no signs of rupture), partial rupture, complete rupture, and avulsion fracture. The Regan and Morrey classification (Regan and Morrey 1989) was used for classification of coronoid fractures. A type-I fracture is an avulsion fracture, a type-II fracture consists of < 50% of the coronoid height, and a type-III fracture of consists of > 50% of the coronoid height. In cases of doubt when analyzing the MR images, the final decision was made by a single musculoskeletal radiologist (JT).

## Results

On conventional radiographs, a Mason type-I fracture was found in 17 elbows, a Mason type-II fracture in 23 elbows, and a Mason type-III fracture in 6 elbows. 2 patients, both with a Mason type-III fracture, presented with a posterior dislocation of the elbow. With MRI, 2 elbows with a Mason type-II fracture were classified as a Mason type-I fracture. Associated osseous injury on conventional radiographs was diagnosed in 3 elbows: 1 fracture of the coronoid process, 1 scaphoid fracture, and 1 avulsion fracture of the lateral epicondyle.

In 35 of the 46 elbows, additional concomitant injuries were diagnosed with MR imaging of the elbow (Table 1). 11 of the 17 elbows with a Mason type-I fracture had associated injury: 8 elbows had injury to the LCL and 8 elbows had capitellar injury. 17 of the 23 elbows with a type-II fracture had an associated injury: 15 elbows had LCL injury and 8 elbows had capitellar injury (Figure). 2 elbows had a loose body and 1 elbow had bone bruising of the lateral epicondyle. All 6 elbows with a Mason type-III fracture suffered associated injuries: 5 elbows had LCL injury and 1 had a rupture of the MCL. There was no reliable observation of ligamentous injuries because of movement artefacts in 1 elbow with a Mason type-III fracture. Osteochondral damage to the capitellum was seen in 2 elbows. 1 elbow with a Mason type-III fracture had a coronoid fracture. 1 patient underwent surgery of the injured elbow: an open reposition and internal fixation of the comminuted fracture of the radial head and a refixation of the LCL. The findings with the MR scan were confirmed during surgery—except for a complete tear of the LCL, which had been diagnosed as a partial tear by MRI (Table 2).

**Table 1.** Details of all the elbows in the series

A	B	C	D	E	F	G	H	I	J	K	L	M
1	1	+	29	1	5	–	–	–	–	–	–	–
2 ^a^	1	–	28	1	4	–	1	3	–	–	–	–
3	0	+	44	1	15	–	–	1	–	–	–	–
4	1	–	51	1	3	–	1	–	–	–	–	–
5	0	–	45	1	2	–	–	–	–	–	–	–
6	0	–	21	1	5	–	–	1	–	–	–	–
7	1	+	61	1	5	–	2	–	–	–	–	–
8	0	+	45	1	7	–	1	–	–	–	–	–
9	0	+	26	1	6	–	–	1	–	–	–	–
10	0	–	56	1	8	–	1	–	–	–	–	–
11	1	–	55	1	1	–	1	2	–	–	–	–
12	0	+	42	1	3	–	–	–	–	–	–	–
13	0	+	52	1	5	–	1	2	–	–	–	–
14	1	+	29	1	8	–	1	1	–	–	–	–
15 ^b^	0	+	44	1	9	–	–	–	–	–	–	–
16	1	+	20	1	6	–	–	1	–	–	–	–
17	1	–	60	1	11	–	–	–	–	–	–	O
18	0	–	48	2	9	–	–	–	–	–	–	–
19	1	+	35	2	10	–	2	2	–	–	1	–
20	0	–	45	2	10	–	2	–	–	–	–	–
21	0	+	53	2	16	–	–	2	–	–	–	–
22	1	+	53	2	10	–	–	2	–	–	–	–
23	0	+	75	2	6	–	2	3	–	–	–	–
24	1	–	37	2	13	–	0	–	–	–	–	–
25	1	–	35	2	13	–	–	–	–	–	–	–
26	1	+	22	2	13	–	0	–	–	–	–	S
27	0	–	60	2	2	–	0	–	–	–	–	–
28	0	–	61	2	1	–	1	1	–	–	–	–
29 ^b^	0	–	44	2	9	–	2	–	–	–	1	–
30	0	–	25	2	7	–	1	–	–	–	–	–
31	0	+	36	2	3	–	–	–	–	–	–	–
32	0	–	38	2	1	–	–	–	–	–	–	–
33	0	+	41	2	2	–	1	–	–	–	–	–
34	0	–	69	2	3	–	0	–	–	–	–	–
35 ^a^	1	+	28	2	4	–	–	–	–	–	–	–
36	1	+	59	2	8	–	1	–	–	–	–	–
37	1	+	37	2	16	–	0	2	–	–	–	O
38	1	+	35	2	9	–	–	1	–	–	–	–
39	1	+	54	2	7	–	2	1	–	–	–	–
40	0	–	34	2	4	–	–	–	–	–	–	–
41	0	+	53	3	6	–	3	–	–	–	–	–
42	0	+	68	3	10	2	2	–	–	1	–	–
43	1	+	45	3	11	–	1	1	–	–	–	M
44	0	+	51	3	4	–	1	–	–	1	–	Su
45	0	–	64	3	6	x	x	–	1	–	–	M
46	0	–	52	3	6	–	2	3	–	–	–	–

^**a, b**^ bilateral fracture of the radial head in two patients.

A case B sex  0 female  1 male C dominant side D age (years) E Mason-Hotchkiss type F number of days between trauma and MRI G MCL lesion  0 contusion  1 partial  2 complete  3 avulsion fracture H LCL lesion  0–1, see G I capitellum  1 bone edema  2 chondral damage  3 fracture J coronoid fracture (Regan and Morrey classification)  1 type-I  2 type-II  3 type-III K dislocation of the elbow joint L loose body M other  M movement artefacts  O edema of lateral epicondyle  S scaphoid fracture  Su elbow surgery

**Figure:**
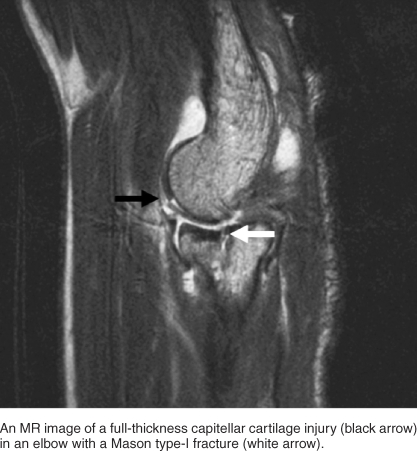
An MR image of a full-thickness capitellar cartilage injury (black arrow) in an elbow with a Mason type-I fracture (white arrow).

**Table 2.** The number of elbows with a Mason type-I, type-II, and type-III fracture and associated injuries on MR imaging

Associated injury	Mason type		
	I (n = 17)	II (n = 23)	III (n = 6)
LCL	8	15	6
MCL	–	–	1
Capitellar injury	8	8	2
Loose osteochondral fragment	–	2	–
Bone bruise lateral epicondyle	–	1	–
Coronoid fracture	–	1	
Any type of associated injury	12	17	6

## Discussion

Diagnosis of associated soft tissue injuries of the elbow with MR imaging can be difficult, but ligamentous structures of the elbow can be evaluated with good sensitivity and specificity (Potter et al. 1997, Hill et al. 2000, Kaplan and Potter 2006). Inter-observer reliability of diagnosing LCL lesions with MR imaging has been graded moderate to good (Carrino et al. 2001). Mirowitz and London (1992) found a high correlation between abnormalities of the MCL seen on MR imaging and pathological findings. Itamura et al. (2005) described a coefficient of variation of less than 5% for intra-observer reliability. The F-test between two observers was not statistically significant for each MR imaging set. However, our study is limited, as the inter- and intra-observer reliability and the clinical relevance were not established.

We found that MR imaging of patients with a radial head fracture revealed associated injuries in three-quarters of them, thus supporting recent studies. These injuries may be an explanation for long-standing symptoms after an adequate treatment of radial head fracture (Davidson et al. 1993, van Riet et al. 2005, Itamura et al. 2005). Early diagnosis of these injuries using MR imaging may contribute to a better understanding of the injuries of the patient with a radial head fracture, thus optimizing (surgical) treatment and giving better outcome.

The LCL typically ruptures as a result of external rotational forces and valgus moment under axial load after a fall on the outstretched hand. If the rotational forces continue, a dislocation can finally occur, with or without rupture of the MCL. An elbow dislocation can also occur without rupture of the MCL (O'Driscoll et al. 1992). Because of the axial loading of the forearm, the radiocapitellar joint is forcefully compressed. This explains the high incidence of associated capitellar injury. Fractures of the coronoid process are common in posterior (sub)luxations of the elbow, as the valgus force under axial load pushes the tip of the coronoid away against the trochlea. Hausman et al. (2009) found partial disruptions of the interosseous membrane in 9 of 14 patients with Mason type-I radial head fractures using MR imaging. None of these disruptions were of clinical importance (Hausmann et al. 2009). As the entire forearm was not scanned in our study, we cannot draw any conclusions about injury to the interosseous membrane.

39% of the patients were reported to have concomitant fractures or clinically significant soft tissue injury in a large retrospective study of 333 adults with radial head fractures (van Riet et al. 2005). LCL insufficiency occurred in 11% and MCL insufficiency in 2%. Combined lesions of the LCL and MCL occurred in 6%. Mason type-I radial head fractures are likely to be stable fractures, without any ligamentous injury. Mason type-II and type-III radial head fractures are frequently associated with ligamentous injury and other fractures (Davidson et al. 1993, van Riet et al. 2005, Doornberg et al. 2007). We found LCL injury in 8 of 17 Mason type-I fractures. Probably not all ligamentous lesions detected with MRI are of clinical importance, e.g. ligamentous distorsion or partial lesions, where stability remains intact.

The incidence of clinically relevant osteochondral lesions of the capitellum is reported to be 2% of all radial head fractures (van Riet et al. 2005). In patients with Mason type-II and type-III fractures who have undergone elbow surgery, the incidence rises to 14–20% (Michels et al. 2007, Nalbantoglu et al. 2009). Itamura et al. (2005) found osteochondral defects in one-third of 24 patients with Mason type-II and type-III fractures using MR imaging (Itamura et al. 2005). We found osteochondral defects in 6 of 46 elbows. Osteochondral damage is possibly the least recognized associated injury in patients with a radial head fracture. Cartilagenous damage is usually not visible on conventional radiographs, but may cause persistent symptoms such as crepitus and mechanical elbow locking.

The incidence of associated injuries of the elbow with radial head fractures by MR imaging is high. The clinical importance of these associated injuries found with MR imaging should be investigated. The treating physician should be aware of the associated injuries and must take these into account when treating patients with radial head fractures. Persistent pain or other symptoms after a radial head fracture may have causes other than the fracture itself.
